# Comparison of daily step count between the Fitbit Inspire 3 and the activPAL 3 in adults with transtibial amputation

**DOI:** 10.3389/fresc.2024.1331005

**Published:** 2024-02-07

**Authors:** Kyle R. Leister, Sara E. Burke, Joon Young Kim, Victor H. Duenas, Tiago V. Barreira

**Affiliations:** ^1^ Department of Clinical and Rehabilitative Sciences, East Tennessee State University, Johnson City, TN, United States; ^2^ Department of Exercise Science, Syracuse University, Syracuse, NY, United States; ^3^ Department of Psychology, Syracuse University, Syracuse, NY, United States; ^4^ Department of Mechanical and Aerospace Engineering, Syracuse University, Syracuse, NY, United States

**Keywords:** physical activity, fitbit, activPAL, amputation, steps, validity

## Abstract

**Introduction:**

Physical activity has significant positive effects on health. Accelerometers can be used to track daily physical activity. The Fitbit Inspire 3 is a commercially available health and fitness tracker, but its validity for tracking steps among individuals with transtibial amputation has not been examined. Therefore, the purpose of this study was to evaluate the concurrent validity of the Fitbit Inspire 3 for assessing free-living daily steps in adults with transtibial amputation.

**Methods:**

Participants (*n* = 79) completed a general health survey and were provided with a Fitbit Inspire 3 and activPAL 3 accelerometer to wear concurrently for seven days in their home environment. Relationships between the activPAL and Fitbit Inspire 3 were examined using Pearson's Correlation. Paired samples t-tests, mean difference, mean absolute difference, and equivalence testing were used to compared daily step counts between Fitbit Inspire 3 and activPAL 3.

**Results:**

Average step counts were 5,768 ± 3,750 (mean ± SD) and 4,674 ± 3,081 by the Fitbit Inspire 3 and activPAL, respectively. A high correlation (*r *= 0.93) but significant mean difference was found between the activPAL 3 and Fitbit Inspire 3 (*p* < 0.001). The mean absolute difference between the devices was 1,347 ± 1,184 steps. On average, the Fitbit Inspire 3 counted 1,094 ± 1,423 more daily steps than the activPAL 3. Equivalency could not be claimed between the devices.

**Discussion:**

The Fitbit Inspire 3 counted more steps compared to the activPAL. Because of the significant mean differences and the large mean absolute difference between the devices, the activPAL 3 and Fitbit Inspire 3 are not interchangeable for estimating physical activity in individuals with transtibial amputation. However, due to the high correlation, the devices will produce similar classification rankings based on step counts.

## 1 Introduction

Mobility and physical activity (PA) influence one's functional status, which is a primary determinant of independence and quality of life (1). This is especially true among lower extremity prosthesis users, who often present with reduced mobility post-amputation. After lower extremity amputation, mobility limitations inherent with limb loss and prosthesis use typically manifest. Given these challenges, several studies have reported decreased PA among individuals with amputation (2, 3).

Decreased PA among this population is problematic because it may result in increased sedentary time and the development of additional comorbidities. As such, assessing daily PA and mobility within the home environment is a clinically relevant objective. Accurately measuring PA may help to identify an individual with transtibial amputation (TTA) who may be at risk for further health deterioration after surgery. This information may also inform prosthetic rehabilitation efforts.

Accelerometers are simple, innocuous, wearable devices that can be used to monitor daily levels of ambulatory PA, and, therefore, represent feasible tools for assessing physical behaviors (4). A device's cost, availability, and ease of use should be considered when selecting an accelerometer to monitor PA in special populations, including those with an amputation. In addition, the device's concurrent validity should be evaluated before interpreting data output that may be used to inform clinical decisions.

Concurrent validity is a subtype of criterion validity that assess the extent of the agreement between two measurements taken simultaneously (5). The primary objective of concurrent validity is to compare the results of a new device or measurement instrument with those of an already established criterion (6). Concurrent validity is an important aspect of psychometric evaluation that provides evidence for the accuracy and effectiveness of the new measurement instrument compared to an established criterion measure. Based on these factors, investigating concurrent validity is an important objective and should be prioritized.

The activPAL 3 is a thigh-worn, research-grade accelerometer that has been extensively used to measure physical behaviors and has demonstrated strong validity in capturing walking, sedentary behavior, and sleep activity measurements in adults (7, 8). The activPAL has also been used in studies featuring individuals with amputation (9–11). Deans et al. assessed the criterion-related validity of the activPAL for measuring various step parameters among a group of adults with unilateral lower extremity amputation (12). In the study, the activPAL’s validity was compared with direct observation of steps taken during a series of laboratory-based tasks. Findings supported that the activPAL was a valid instrument for detecting purposive stepping among prosthesis users within a laboratory setting.

While the activPAL has been used in various studies featuring individuals with amputation, the validity of the commercially available Fitbit Inspire 3 has not been extensively tested in this group. The Fitbit Inspire 3 is a wrist-worn health and fitness tracker that can be purchased at many commercial retailers, making it more accessible to the general public than research-grade devices such as the activPAL. In addition to greater accessibility, the Fitbit Inspire 3 is water-resistant and less costly than many research-grade wearables. These features make the Fitbit Inspire 3 a more attractive option for individuals with amputation who are interested in monitoring their daily PA.

The Fitbit Charge 2, Fitbit Ultra, and Fitbit Inspire HR have been validated in various clinical populations, but step count accuracy assessment is currently limited among individuals with TTA (13–15). Assessing the Fitbit Inspire 3's concurrent validity among this group is essential because The Fitbit Inspire 3 represent a more feasible, cost-effective, and intuitive option for clinicians to assess rehabilitative outcomes outside of the clinical setting. The Fitbit Inspire 3 may also serve as a motivation tool for a prosthesis user interested in enhancing their daily PA.

Considering these potential benefits, this study aimed to investigate the concurrent validity of the Fitbit Inspire 3 for assessing free-living daily step count among individuals with TTA. To address this aim, daily step data collected via the Fitbit Inspire 3 were compared with the research-grade activPAL 3 accelerometer in adults with TTA.

## 2 Materials and methods

### 2.1 Participants

The study was conducted according to the Declaration of Helsinki. All participants provided written informed consent in accordance with Syracuse University's Institutional Review Board approved protocol. As part of a larger multicenter study, a cross-sectional design was used to investigate the concurrent validity of the Fitbit Inspire 3 to assess daily steps among individuals with TTA in their free-living environment. All participants were recruited from a network of orthotic/prosthetic clinics across the United States. Inclusion/exclusion criteria were determined after evaluating responses on a self-reported medical history questionnaire.

#### 2.1.1 Inclusion criteria

All participants were between the ages of 18 and 80 and had a unilateral TTA. All participants had used a prosthesis for at least three months before beginning the experimental protocol. It is estimated that 28.2% of amputations occur at the transtibial level, making it the second most common amputation type, trailing only toe/partial foot amputation (33.2%) (16–18). Thus, recruitment was limited to individuals with TTA to increase general applicability and recruitment feasibility.

#### 2.1.2 Exclusion criteria

Participants were provided with a list of movement disorders as part of a comprehensive medical history questionnaire and were asked to identify any movement disorders that may have drastically impacted their mobility (i.e., stroke, Parkinson's disease, spinal cord injury, traumatic brain injury). Participants who self-reported a movement disorder that may have impacted their mobility were excluded. This criterion was established as various movement disorders may further perturb gait biomechanics beyond what is typically noted with prosthesis use, which may confound device validation efforts (19, 20).

### 2.2 Study design

The experimental protocol was initiated during one encounter at the clinic where the participant regularly received prosthetic care. During the encounter, participants completed a general health survey and were provided with activPAL 3 and Fitbit Inspire 3 devices to wear concurrently for seven days in their home environment. Participants were asked to return the devices to the same location or send the devices via the postal service in a self-addressed stamped envelope.

#### 2.2.1 Health screening

Baseline screening information including ethnicity, sex, age, height (measured with a stadiometer), weight (measured with an electronic scale), and BMI were computed for each participant. Participants were then asked specific questions pertaining to their amputation and current prosthesis (cause of amputation, amputation date, years of prosthesis utilization, age of current prosthesis). Information regarding the participant's type 2 diabetes status, including the date of diagnosis and treatment modality, was also collected during the initial screening. Information pertaining to each individual's type 2 diabetes status was collected as type 2 diabetes is the leading cause of all nontraumatic amputations and was therefore considered an important metric in which to classify the sample (21, 22).

#### 2.2.2 activPAL 3 assignment

Each participant was provided with an activPAL 3 (software version 8.11.1.63, analysis algorithm CERA v1.3). Prior to assigning each activPAL, it was visually confirmed in the device’s software suite that each device was using identical software and algorithm versions. The activPAL 3 is a triaxial accelerometer with a sampling frequency of 20 Hz and a dynamic range of ±2 gravitational units (8). The device weighs 20 g (5 cm × 3.5 cm × 0.7 cm) and estimates sitting, standing, walking, and daily steps using proprietary algorithms based on acceleration measurements. The activPAL was attached to the sound side (non-amputated) thigh with Hypafix tape, per Deans et al.'s recommendations (12). The activPAL's validity and accuracy for assessing walking activity among lower extremity prosthesis users has been evaluated and confirmed by Salih et al. (11).

#### 2.2.3 Fitbit Inspire 3 assignment

Each participant was also provided with a Fitbit Inspire 3 (software version 1.188.58). Prior to assigning each Fitbit Inspire 3, it was confirmed that each device was using identical software versions. The Fitbit Inspire 3 is a microelectromechanical triaxial accelerometer that collects data in 60 sec epochs and converts raw acceleration information to step counts using proprietary algorithms. The device weighs 23 g (14 cm × 17.6 cm × 1.4 cm) and measures standard PA metrics, including step count, distance, active minutes, and sleep. Per the manufacturer's recommendation, the Fitbit Inspire 3 was worn on the non-dominant wrist. All devices were linked to a corresponding research account (rather than a personal account) only accessible to the researchers. Participants could, however, track their daily steps by viewing the device's output screen. Daily step count data recorded by the Fitbit Inspire 3 were extracted by logging into the research account and analyzing the software's daily step count log.

Various Fitbit models have been validated for overground walking among special populations. Fulk et al. reported that the Fitbit Ultra was a valid, low-cost option for measuring stepping activity in level, predictable environments for people with stroke (ICC = 0.73) (13). In a second study featuring individuals with obesity, McVeigh et al. found that the Fitbit Charge 2 had high correlation when compared with the ActiGraph GT3X + (*r* = 0.94) for assessing daily steps. These studies suggest that the Fitbit Ultra and Charge 2 may be valid tools for assessing step count in these clinical populations (14).

#### 2.2.4 activPAL 3 and Fitbit Inspire 3 wear protocol

Written and verbal donning/doffing instructions were provided to each participant. Both the Fitbit Inspire 3 and activPAL 3 were simultaneously temporally synchronized. The same computer, power cord, and docking system were utilized to synchronize the devices within their respective software suites. After temporal synchronization, participants donned each device and were instructed to wear both devices at all times for seven days, only removing when in contact with water. A minimum of four days was necessary for participants to be included in the data analysis. activPAL 3 and Fitbit Inspire 3 data were manually matched for waking wear periods according to the activPAL 3 data. Thus, only valid wear time during waking hours simultaneously recorded on both devices was included for statistical analyses. Once the same periods were identified across the same days, each device's average step count value (per day) was compared. The daily step counts from at least four valid days were averaged, resulting in a single step count value for each participant for each device.

### 2.3 Statistical analysis

The relationships between the activPAL 3 and Fitbit Inspire 3 were examined using a Pearson correlation. Based on previously published standards, an observed correlation coefficient between 0.40–0.59, 0.60–0.79, and 0.80–1.00 was considered moderate, moderately high, and high, respectively (23).

A paired samples *t*-test was conducted to identify mean differences between the activPAL 3- and Fitbit Inspire 3-assessed step daily counts. Mean difference and mean absolute difference (MAD) were calculated to determine differences between methods.

Equivalence testing using the confidence interval method was conducted to compare activPAL 3 vs. Fitbit Inspire 3 daily step counts (24). Step values from the Fitbit Inspire 3 were statistically equivalent (at an α = 0.05) if the 95% confidence intervals of the mean step value fell within the equivalence zone. The equivalence zone was set at ±10% of the mean activPAL 3 data.

A Bland-Altman plot was created by adding reference lines to a scatterplot. The mean difference and upper and lower reference lines representing the 95% confidence interval for the measures were represented in the plot. All statistical analyses were conducted using SPSS, and the level of significance was defined as *p* < 0.05.

## 3 Results

A total of 79 adults with TTA (58.1 ± 14.8 years; mean ± SD; 22 females) provided valid Fitbit Inspire 3 and activPAL 3 data; see Table 1 for summary demographics. A high correlation was found between the devices (*r* = 0.93) (Table 2). However, the paired samples *t*-test revealed a significant mean difference (*t*_78_ = −6.83, *p* < 0.001) (Table 2). The activPAL 3 estimated an average of 4,674 ± 3,081 daily steps, whilst the Fitbit Inspire 3 estimated 5,768 ± 3,750 daily steps. The mean difference and MAD between the activPAL 3 and Fitbit Inspire 3 were −1,094 ± 1,423 and 1,347 ± 1,184 steps, respectively (Table 2).

**Table 1:** Demographic and clinical characteristics of participants (mean ± SD).

Characteristic	Value
Total sample age (Years)	*n* = 79 58.1 ± 14.8
Sex	22 female
Ethnicity	
Asian	2
Black or African American	12
Hispanic or Latino/a	2
White	63
Amputation cause	
Vascular Disease/Diabetes	39
Injury/Trauma	26
Infection (Without diabetes)	7
Cancer/Tumor	3
Congenital/Birth	3
Other	1
Body mass index	30.7 ± 6.0
Years of prosthesis utilization	11.8 ± 13.9
Age of current prosthesis	2.13 ± 1.9

**Table 2:** Analysis results for activPAL 3 and Fitbit Inspire 3 daily step count (mean ± SD).

Device	Mean step counts per day	Difference (steps)	Absolute difference (steps)	Correlation	t	*p*
activPAL 3	4,674 ± 3,081	−1,094 ± 1,423	1,347 ± 1,184	0.93	−6.83	<.001
Fitbit Inspire 3	5,768 ± 3,750					

The 95% confidence interval for the discrepancy between the Fitbit Inspire 3 and activPAL fell entirely outside of the previously specified interval for equivalency, indicating that equivalency could not be claimed (lower 95% confidence interval: *t*_78_ = 9.75, *p* < 0.001; upper 95% confidence interval: *t*_78_ = 3.91, *p* > 0.99).

**Figure 1:**
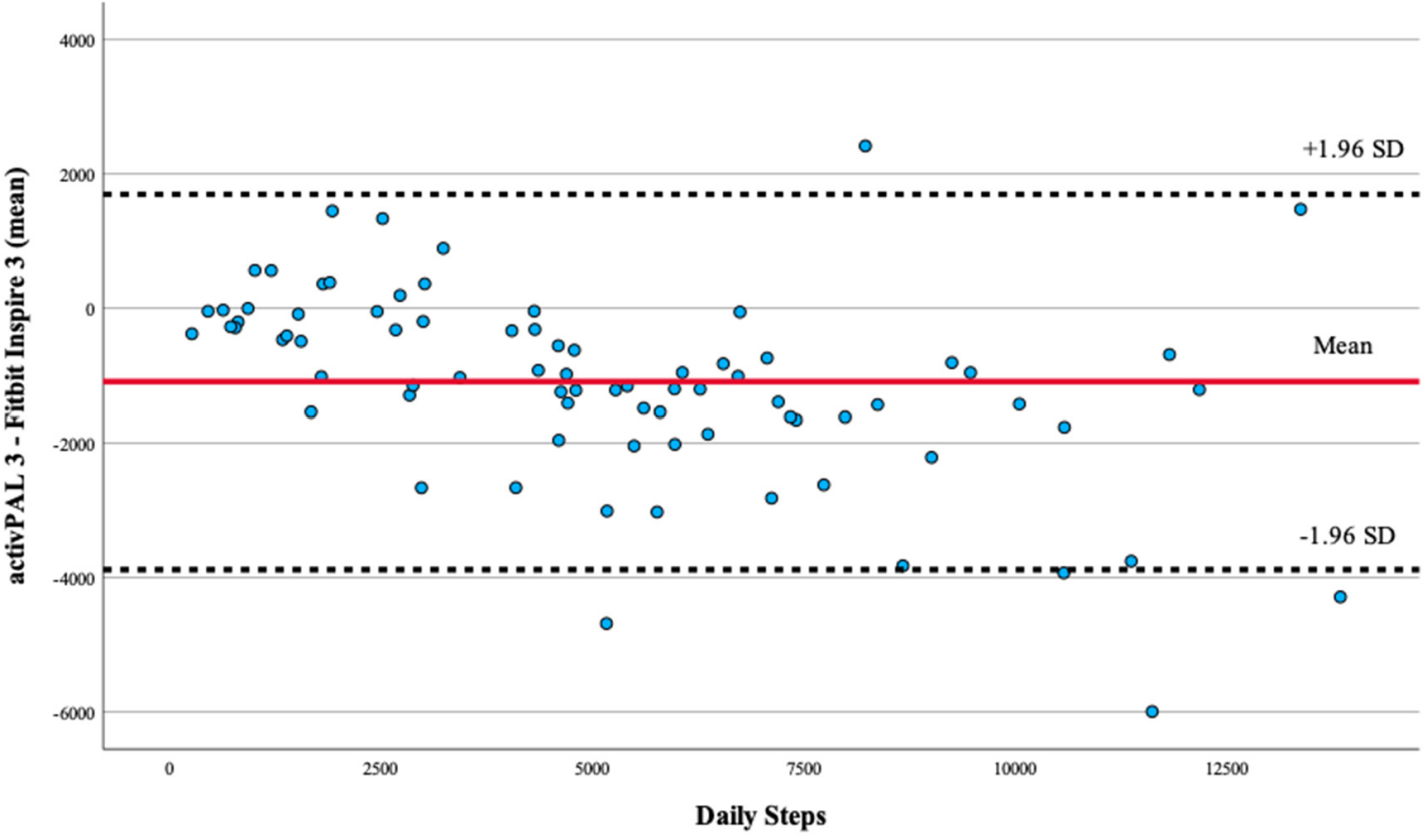
Bland–Altman plot of activPAL 3- and Fitbit Inspire 3-counted daily step count values. Bland–Altman plots comparing activPAL vs. the Fitbit Inspire 3 yielded four participant data points outside the 95% limit of agreement (±1.96 SD).

Bland-Altman plots comparing activPAL 3 to the Fitbit Inspire 3 yielded four data points outside the 95% limit of agreement (±1.96 SD) (Figure 1).

## 4 Discussion

Regular PA is an important component of health and well-being, particularly in individuals who present with decreased mobility, such as individuals with TTA (2, 25). Accurately measuring PA is fundamental for evaluating a rehabilitative intervention's effectiveness and understanding mobility's impact on health outcomes. In this study, the concurrent validity of the Fitbit Inspire 3 health and fitness tracker step counts measure was evaluated. The activPAL 3 and Fitbit Inspire 3 were highly correlated, indicating that both devices are related and capable of measuring similar constructs. However, the statistically significant paired samples *t*-test and large mean difference and MAD between the devices indicate that the activPAL 3 and Fitbit Inspire 3 may not be interchangeable for measuring free-living daily steps for individuals with TTA.

The Fitbit Inspire 3 recorded an average of 1,094 more daily steps than the activPAL 3, suggesting it may be more sensitive when capturing steps. These findings imply that while both devices can measure PA, caution should be exercised when comparing step count data between the activPAL 3 and Fitbit Inspire 3 to inform clinical decisions.

The discrepancy of 1,094 steps represents a 23% difference between the two devices. When contextualizing this difference within the framework of established benchmarks for clinical significance, a 10% difference has conventionally been considered acceptable (26). However, it is crucial to recognize that the interpretation of what constitutes a clinically meaningful difference can vary based on the population's specific characteristics and the nature of the outcome measure.

From a clinical standpoint, a meta-analysis conducted by Kang et al. concluded that a 2,600 step per day increase may be expected with accelerometer-based PA interventions among healthy individuals without amputation (27). Applying this comparison to individuals with TTA is challenging given wide variability in daily steps among this population. Further, the relationship between health outcomes and daily step count remains unclear for individuals with TTA. Nevertheless, considering the percentage difference and observed improvements in interventions with accelerometers, a 1,094-step disparity may indeed be noteworthy.

The lack of equivalency between the devices also highlights the importance of selecting the appropriate device for individuals with TTA. While the Fitbit Inspire 3 may be a more user-friendly, cost-effective option, it does not appear to provide comparable estimates to the activPAL for this group. Clinicians should consider these differences when selecting an appropriate device for patients interested in monitoring their daily PA, as measurement inaccuracies could impact treatment outcomes.

One possible explanation for the observed differences may be attributed to each device's anatomical placement. In the current study, the Fitbit Inspire 3 was worn on the non-dominant wrist, while the activPAL 3 was worn on the thigh of the non-amputated limb. Although wrist-worn devices are popular for monitoring daily steps due to their convenience and wide availability, they may overestimate steps in certain situations, such as when the arms are moving and the lower extremities are stationary or when an individual is handling or manipulating objects while in a seated or static standing position (28–31). These phenomena are highlighted by Nelson et al., who reported that wrist-worn accelerometers can overestimate steps during free-living conditions by 10%–35% when compared to devices worn on the lower body (31). In contrast, thigh-worn devices are less prone to such inaccuracies, as the lower extremity typically accelerates only during ambulatory activities (6, 32).

These observations are also supported by Montoye et al., who found that thigh-worn accelerometers more accurately predicted light- and moderate-intensity PA and sedentary behavior compared to wrist- and hip-worn devices (33). In the study, participants completed three sedentary and 10 non-sedentary activities for 3–10 min each. Direct observation was used as the criterion measure of each activity, and a machine learning model was created for each accelerometer to predict the PA intensity category. The sensitivity and specificity were higher for the thigh-worn device compared to the wrist- and hip-worn accelerometers (>99%). Ultimately, the thigh-worn device provided a more accurate PA assessment under all conditions, while all other accelerometers overestimated PA.

Biomechanical differences often observed among individuals with TTA may offer a second potential explanation for the discrepancies noted between the devices. It widely accepted that individuals with TTA face unique challenges, including decreased mobility, increased energy expenditure, and altered gait biomechanics (34–36). Individuals with TTA often walk at a slower cadence than healthy individuals, which may exacerbate discrepancies between wrist- and thigh-worn devices (37). Hermodsson et al. reported that individuals with TTA secondary to vascular and traumatic etiology had significantly reduced walking speeds compared to healthy individuals during an overground walking test on an instrumented force platform (vascular: 0.85 ± 0.2 m/s; trauma: 0.99 ± 0.2 vs. healthy: 1.42 ± 0.2 m/s) (37). Given the decreased gait velocities exhibited by individuals with TTA, selecting an accelerometer that can capture slower movement signals is essential. The activPAL has been shown to be superior for capturing steps performed at a slower cadence, which may make it a more accurate option for tracking steps in individuals with TTA (6, 38, 38).

The current study had several strengths including a relatively large sample size (*n* = 79), which permits a more diverse representation within the study group. By including a diverse group of individuals with TTA, the study becomes more generalizable to the broader population of individuals with TTA. This, in turn, enhances the external validity of the research, allowing the findings to be applied to a wider range of individuals with similar characteristics. The study's real-world setting represents a second strength. Capturing daily step count within the participant's home environment enhances the study’s ecological validity, as participants may be more likely to engage in their typical daily activities and routines when monitored at home. This captures an individual’s natural behavior, providing a more accurate representation of their mobility profile.

Although carefully conducted, there are noteworthy limitations to the current study. One potential limitation is that the sample was only comprised of individuals with TTA. Future studies featuring individuals with amputations at other levels (transfemoral, hip disarticulation, etc.) are needed to determine the accuracy and equivalency of the activPAL 3 and Fitbit Inspire 3 for individuals with amputation levels other than transtibial. The study did not explore the potential factors that could contribute to the differences in step count estimates between the two devices, such as differences in placement, attachment, or algorithm sensitivity. Future studies should be conducted to examine these factors. Lastly, a direct measure of steps was not utilized amongst the sample and therefore the true daily step counts are unknown. While direct measures are not always feasible for all free-living activities, they can provide valuable insights into the device’s accuracy, especially during shorter periods when direct step measurement is reasonable. Despite this limitation within the current study, the activPAL 3 has been shown to be accurate in short bouts of PA in this population in previous research (11, 12).

Overall, the findings of this study suggest that individuals with TTA should be cautious when selecting and interpreting data from commercially available wearable activity monitors. Although these devices can be valuable tools for monitoring PA and tracking mobility progress, inter-device comparisons may be nuanced and not always provide accurate and/or interchangeable data. This study highlights the importance of acknowledging the incongruities between commercially available and research-grade accelerometers.

## 5 Conclusions

The present study provides important insights into the validity of the Fitbit Inspire 3 for estimating step count for individuals with TTA. While a strong relationship was found between the activPAL 3 and Fitbit Inspire 3, the Fitbit Inspire 3 likely counted more daily steps relative to the research-grade activPAL 3, indicating that the devices may not be equivalent or interchangeable in this population. Therefore, researchers and clinicians should consider these findings when selecting a device to monitor step count for individuals with TTA and interpreting data obtained from the Fitbit Inspire 3.

## Data Availability

The raw data supporting the conclusions of this article will be made available by the authors, without undue reservation.
